# Mitochondrial DNA Pathogenic Variants in Ophthalmic Diseases: A Review

**DOI:** 10.3390/genes16030347

**Published:** 2025-03-17

**Authors:** Khaled K. Abu-Amero, Bashaer Almadani, Shereen Abualkhair, Syed Hameed, Altaf A Kondkar, Andrea Sollazzo, Angeli Christy Yu, Massimo Busin, Giorgio Zauli

**Affiliations:** 1 Research Department, King Khaled Eye Specialist Hospital, Riyadh 11462, Saudi Arabia; bmadani@kkesh.med.sa (B.A.); sabualkhair@kkesh.med.sa (S.A.); syhameed@kkesh.med.sa (S.H.); gzauli@kkesh.med.sa (G.Z.); 2 Department of Ophthalmology, College of Medicine, King Saud University, Riyadh 11411, Saudi Arabia; akondkar@ksu.edu.sa; 3 Department of Translational Medicine, University of Ferrara, 44121 Ferrara, Italy; sllndr@unife.it (A.S.); angelichristy.yu@unife.it (A.C.Y.); massimo.busin@unife.it (M.B.)

**Keywords:** DNA pathogenic variant, mitochondria, ophthalmology

## Abstract

Mitochondria are vital organelles responsible for ATP production and metabolic regulation, essential for energy-intensive cells such as retinal ganglion cells. Dysfunction in mitochondrial oxidative phosphorylation or mitochondrial DNA (mtDNA) pathogenic variants can disrupt ATP synthesis, cause oxidative stress, and lead to cell death. This has profound implications for tissues such as the retina, optic nerve, and retinal pigment epithelium, which are dependent on robust mitochondrial function. In this review, we provide a comprehensive compilation of pathogenic variants in the mtDNA associated with various ophthalmic diseases, including Leber’s hereditary optic neuropathy, chronic progressive external ophthalmoplegia, Leigh syndrome, mitochondrial encephalomyopathy, lactic acidosis, and stroke-like episodes, among others. We highlight the genetic variants implicated in these conditions, their pathogenic roles, and the phenotypic consequences of mitochondrial dysfunction in ocular tissues. In addition to well-established mutations, we also discuss the emerging evidence of the role of mtDNA’s variants in complex multifactorial diseases, such as non-arteritic anterior ischemic optic neuropathy, primary open-angle glaucoma, and age-related macular degeneration. The review aims to serve as a valuable resource for clinicians and researchers, providing a detailed overview of mtDNA pathogenic variants and their clinical significance in the context of mitochondrial-related eye diseases.

## 1. Introduction

Mitochondria are essential subcellular organelles in eukaryotes, often referred to as the powerhouse of the cell. Mitochondria are a primary site of ATP synthesis and play a key role in regulating metabolism to provide energy for various cellular processes [1,2]. Cells with high metabolic activity, such as retinal ganglion cells (RGCs) of the eye, cardiac muscle, and tissues like the liver, kidney, and muscles, heavily depend on these organelles to meet their energy requirements [3,4]. Consequently, these tissues are highly vulnerable to mitochondrial dysfunction, making them particularly susceptible to diseases caused by ATP disruption [5,6].

Mitochondria produce ATP through oxidative phosphorylation (OXPHOS), involving five respiratory chain complexes (I–V). These complexes oxidize vital molecules, such as NADH and FADH2, to drive ATP production. However, mitochondria are involved in many other essential functions, including fatty acid oxidation, detoxifying reactive oxygen species (ROS), and regulating apoptosis. Defects in these processes, especially in OXPHOS, can cause impaired ATP production, increased oxidative stress, and cell death [7,8]. Mammalian mitochondria contain their own DNA and are inherited maternally. Human mitochondrial (mtDNA) is remarkably small, consisting of 16,569 bases [9]. Pathogenic variants in mtDNA can exacerbate ATP impairments and lead to many diseases, including those affecting the eyes [5,6,9,10].

The human eye, particularly the retina and optic nerve, is among the most metabolically active tissues in the body and is highly dependent on a constant supply of ATP for functions like photoreceptor maintenance and visual signal transmission. In recent years, a growing body of research has demonstrated the involvement of mtDNA pathogenic variants in various ophthalmic diseases [2,10]. Pathogenic variants in mtDNA can severely disrupt mitochondrial function, causing vision-related disorders. These include conditions like Leber’s hereditary optic neuropathy (LHON), Leigh syndrome (LS), neuropathy, ataxia, retinitis pigmentosa (NARP), chronic progressive external ophthalmoplegia (CPEO), Kearns-Sayre syndrome (KSS), mitochondrial encephalomyopathy, lactic acidosis, and stroke-like episodes (MELAS), myoclonic epilepsy with ragged red fibers (MERRF), non-arteritic anterior ischemic optic neuropathy (NAION), primary open-angle glaucoma (POAG), and age-related macular degeneration (AMD). These disorders share a common feature of mitochondrial dysfunction resulting from the disturbed delicate balance between energy production and oxidative stress. The disruption of OXPHOS, particularly due to pathogenic variants in the mtDNA, impairs ATP production, leading to increased oxidative stress, apoptosis, and progressive damage of the RGCs, the optic nerve, or the retinal pigment epithelium (RPE) [2,10]. While each disease has unique clinical manifestations, the shared involvement of mitochondrial dysfunction underscores the critical role of maintaining mitochondrial health to prevent and treat these eye disorders.

Identifying and understanding mtDNA pathogenic variations is critical in the etiology of eye disorders [10]. Our review aims to describe the reported mtDNA pathogenic (P), likely pathogenic (LP), and unknown significance (VUS) variants associated with ocular diseases. It focuses exclusively on mtDNA variants, excluding variants associated with nuclear gene defects, and seeks to bridge the gap between genetic discoveries and their clinical manifestations in the eye. We searched general biomedical databases (PubMed, Embase, Web of Science, GeneReviews, and Scopus). We also included genetics and genomics databases (OMIM, ClinVar, GenBank, and dbSNP (Single Nucleotide Polymorphism Database)). Additionally, we searched specialized mitochondrial databases (MITOMAP, MitoBreak, MitoMiner, MitoDisease, and MitoVariome). We included all mtDNA variants reported in association with ophthalmic diseases. This review is unique because it includes, for the first time, mtDNA variations that are likely pathogenic based on in silico analysis and the fact that they were not identified in normal controls of similar ethnicity to the disease patients. Additionally, it is the first complete collection of ocular diseases linked to mtDNA pathogenic variants, including secondary variants. The review will provide deeper insights into the role of mtDNA in vision health and present a comprehensive resource to clinicians, scientists, and molecular geneticists for furthering research into diagnosis and therapeutic development in ophthalmic genetics of mitochondrial-related eye diseases.

## 2. Ophthalmic Diseases Caused by mtDNA Pathogenic Variants

### 2.1. Leber’s Hereditary Optic Neuropathy—LHON

LHON is an acute onset rare mitochondrial disorder with a prevalence of approximately 1 in 30,000 to 50,000 individuals worldwide, though it can be higher in certain populations. The disease typically manifests in young adults, with the average age of onset between 15 and 35 years, though cases can occur at any age [11,12].

Specific pathogenic variants in mtDNA cause LHON. Among the many mtDNA pathogenic variants reported in LHON (see Table 1), three primary mtDNA point pathogenic variants (m.3460G>A in *MT-ND1*, m.11778G>A in *MT-ND4*, and m.14484T>C in *MT-ND6*) account for approximately 90% of LHON cases globally, with m.11778G>A accounting for 70% of LHON cases and m.3460G>A being the least prevalent [11]. These pathogenic variants impair the normal function of complex I (NADH dehydrogenase), leading to the selective degeneration of RGCs and the optic nerve. Most of these pathogenic variants are homoplasmic, but heteroplasmy has been reported in 10–15% of LHON pedigrees [11,13]. Several other rare causative pathogenic variants (e.g., m.3700G>A, m.3733G>A, m.14482C>A-G) have also been reported in European LHON patients and are postulated to probably have a synergistic effect with the primary pathogenic variant responsible for LHON [14]. The resulting pathology primarily affects central vision, leading to acute or subacute painless vision loss that often progresses to blindness.

Haplogroup J is overrepresented in LHON cases with T14484C and G11778A pathogenic variants, potentially enhancing LHON expression [15]. Sub-haplogroups J2b and Jc1 may increase pathogenic variant penetrance by affecting complex III functions [16]. However, its influence varies by region, and some studies challenge its role in altering disease onset or visual outcomes [17].

The inheritance of LHON follows a maternal pattern, as mtDNA is inherited solely from the mother. However, the condition shows incomplete penetrance, meaning not all individuals with the pathogenic variant develop symptoms, and factors such as smoking, alcohol use, and other genetic modifiers may influence disease expression [18,19].

From a clinical perspective, LHON is diagnosed based on clinical symptoms, family history, and genetic testing to identify the characteristic mtDNA pathogenic variants. Optical coherence tomography (OCT) often reveals early thinning of the retinal nerve fiber layer, even before significant visual loss occurs. Although there is no cure for LHON, idebenone (trade name, Raxone^®^), a synthetic coenzyme Q10 analogue, is currently the only approved treatment for LHON, targeting mitochondrial dysfunction to improve or stabilize vision in some patients [11,20].

### 2.2. LHON-Plus

LHON-plus is a variant of LHON characterized by extraocular manifestations, including neurological or systemic features such as ataxia, peripheral neuropathy, cardiac abnormalities, and myopathy. The prevalence of LHON-plus is lower than LHON, and its symptoms typically develop after visual loss. The pathophysiology involves widespread mitochondrial dysfunction affecting not only RGCs but also other energy-demanding tissues, such as the brain, muscles, and cardiac cells [11]. Like classical LHON, LHON-plus patients are also associated with mtDNA pathogenic variants, often involving the same primary pathogenic variants (m.3460G>A, m.11778G>A, m.14484T>C) [21]). However, additional mtDNA pathogenic variants may also be involved in causing the extended spectrum of symptoms seen in LHON-plus. In addition, cases of LHON-plus demonstrating complex I respiratory defect with no mtDNA pathogenic variant upon sequencing the entire mitochondrial genome are also reported [22]. These cases could benefit from exome sequencing, as nuclear gene variants could be involved. However, the exact mechanisms of how tissues other than the eyes are selectively affected in LHON-plus remain an interesting area of investigation.

### 2.3. Leigh Syndrome/Leigh Syndrome and Spectrum

Leigh syndrome (OMIM 256000), also known as subacute necrotizing encephalomyelopathy, is a rare, progressive neurodegenerative disorder that typically presents in infancy or early childhood, with an estimated incidence of 1 in 40,000 live births that may vary across populations [23]. The disease manifests with a range of symptoms, including psychomotor regression, peripheral neuropathy, cerebellar ataxia, spasticity, and hypotonia. Ocular manifestations include nystagmus, ophthalmoparesis, and optic atrophy. Biochemical evaluation and specific metabolic profiles suggest impairment in the energy production (OXPHOS) in mitochondria. The diagnosis of mtDNA-LS is established by identifying heteroplasmic or homoplasmic pathogenic variants in 1 of the 15 mtDNA genes [24].

Mitochondrial pathogenic variants frequently implicated in Leigh disease include those in the *MT-ATP6* gene, which affects the complex V of the electron transport chain and results in a loss of ATP-synthetic activity. Notably, the m.8993T>G/C pathogenic variants in the *MT-ATP6* gene lead to a severe reduction in ATP production and energy failure, which are central to the disease pathology [25,26]. Other mtDNA pathogenic variants affecting complex I (NADH dehydrogenase), such as pathogenic variants in the *MT-ND3* gene (e.g., m.10191T>C), *MT-ND5* (e.g., m.13513G>A), and *MT-ND6* (e.g., m.14459G>A), also contribute to Leigh syndrome [23]. In addition, pathogenic variants in mtDNA-encoded tRNA genes, such as m.3243A>G tRNA-Leu and m.8363G>A tRNA-Lys and G14459A and T14484C point pathogenic variants [27]. The most widespread mtDNA pathogenic variant in Leigh syndrome is at mtDNA, 8993T>G/C, affecting the ATP6 subunit of the ATPase, and is also associated with maternally inherited Leigh syndrome (MILS), as well as neuropathy, ataxia, and retinitis pigmentosa (NARP) [23,24]. LS, with its wide spectrum of phenotypes, is also associated with many other nuclear gene variants.

### 2.4. Maternally Inherited Leigh Syndrome—MILS

Maternally inherited Leigh syndrome (MILS) is a severe neurodegenerative disorder clinically characterized by psychomotor delay, muscle weakness, developmental regression, and bilateral symmetrical lesions in the basal ganglia, which are characteristic of the disease [24]. Respiratory issues, difficulty feeding, movement disorders, and seizures are often the common presentations in these patients. As the disease progresses, it affects the brainstem, leading to respiratory failure and death. The prognosis is poor, with most patients succumbing to the disease within a few years of onset [23,28]. Unlike Leigh syndrome, MILS is exclusively maternally inherited due to its basis in mtDNA pathogenic variants, and 100% of cases are due to mtDNA pathogenic variants, specifically in the *MT-ATP6* gene. The most common mtDNA pathogenic variant associated with MILS is m.8993T>G. Another pathogenic variant, m.8993T>C, also affects the same region but may present with a milder phenotype depending on the degree of heteroplasmy [23,24,29].

### 2.5. Neuropathy, Ataxia, and Retinitis Pigmentosa—NARP

NARP syndrome, characterized by neuropathy, ataxia, and retinitis pigmentosa, is primarily linked to pathogenic variants in mtDNA, particularly in the *MT-ATP6* gene [30]. Studies have identified various pathogenic variants, including m.8993T>G/C [31], m.5789T>C tRNA cysteine [32], and m.8729G>A [33] in NARP. m.8993T>G is the most common pathogenic variant in the *MT-ATP6* gene, associated with retinal abiotrophy and maternal inheritance; m.5789T>C disrupts the stability of mitochondrial tRNA for cysteine; and m.8729G>A is another novel pathogenic variant linked to classical NARP symptoms [31,34]. Another pathogenic variant, m.9185T>C, in the *ATP6* gene, is associated with a rare mitochondrial disorder, presenting with symptoms resembling NARP and Leigh syndrome [31,34]. These pathogenic variants can result in various clinical manifestations, including nystagmus, hearing loss, ophthalmoplegia, cardiac conduction defects, anxiety, dementia, sleep apnea, short stature, muscle weakness, learning disability, developmental delay, ataxia, seizure, migraine, and sensory neuropathy. This underscores the importance of genetic testing for accurate diagnosis. The presence of elevated lactate and pyruvate levels in patients can indicate mitochondrial dysfunction. Brain imaging may show non-specific cerebral or cerebellar atrophy in patients with high disease burden. While mitochondrial pathogenic variants are central to NARP syndrome, the complexity of symptoms and the potential for overlapping conditions necessitate a comprehensive approach to diagnosis and management. Understanding these pathogenic variants can also inform therapeutic strategies, such as substrate-level phosphorylation enhancement [35].

### 2.6. Chronic Progressive External Ophthalmoplegia (CPEO) and CPEO Plus

CPEO is primarily characterized by a progressive weakness of the extraocular muscles, leading to bilateral symmetric, painless, and pupil-spared ptosis and ophthalmoplegia, causing difficulties with eye movement and an impaired ability to gaze in different directions. CPEO can occur in isolation or with other systemic symptoms in what is referred to as CPEO-plus. In CPEO-plus, patients exhibit additional clinical manifestations beyond the eye muscles, including proximal muscle weakness, exercise intolerance, dysphagia, sensorineural hearing loss, cardiac conduction defects, diabetes, and ataxia [36].

CPEO and CPEO-plus typically begin in early adulthood but can manifest at any age and are primarily associated with pathogenic variants in the mtDNA. The common mtDNA pathogenic variants linked to CPEO include large-scale mtDNA deletions, particularly single deletions in OXPHOS genes, leading to extraocular muscle weakness [37,38]. The single 4977 bp deletion, known as the “common deletion”, is the most frequent genetic defect found in patients with CPEO [39,40]. Pathogenic variants in the *MT-TL1* gene (encoding mitochondrial tRNA leucine), such as the m.3243A>G pathogenic variant, are often found in patients with CPEO, particularly in those with multisystem involvement (CPEO-plus) [41,42]. Pathogenic variants in other mitochondrial tRNA genes, like *MT-TA* (tRNA Ala) [43], *MT-TN* (tRNA Asn) [44], *MT-TI* (tRNA Ile) [45], and *MT-TS* (tRNA Ser) [46], have also been reported in CPEO and CPEO-plus cases.

The severity and progression of CPEO and CPEO-plus are significantly influenced by the degree of heteroplasmy level (proportion of mutated mtDNA) within the affected tissue. Understanding these mtDNA pathogenic variants and their effects on mitochondrial function is critical for effectively diagnosing and managing CPEO and CPEO-plus.

### 2.7. Kearns-Sayre Syndrome—KSS

Kearns-Sayre Syndrome (KSS) classically presents a triad of symptoms: progressive external ophthalmoplegia (PEO), bilateral pigmentary retinopathy, and onset before the age of 20. The additional presence of a cardiac conduction defect, cerebellar ataxia, or an increased cerebrospinal fluid protein level (>100 mg/dL) is essential to make the diagnosis of KSS [47,48]. PEO leads to drooping eyelids and limited eye movement, while pigmentary retinopathy causes progressive loss of peripheral and night vision. Dilated fundus examination reveals diffuse stippled areas of hypopigmentation of the retinal pigment epithelium. Other common features include bilateral symmetrical ptosis, short stature, hearing loss, and endocrine problems such as diabetes and hypothyroidism, reflecting mitochondrial dysfunction in high-energy-demand tissues like the eyes, heart, and muscles. KSS is exceedingly rare, with an estimated prevalence of 1 to 3 cases per 100,000 individuals [49]. The exact incidence of KSS remains unclear due to underdiagnosis, especially in cases with mild symptoms or overlapping features with other mitochondrial disorders.

KSS is caused primarily by large-scale deletions in mtDNA, typically spanning from 1.1 to 10 kb [49,50]. Specific deletions frequently found in KSS patients involve the loss of genes such as *MT-ND1*, *MT-ND4*, *MT-ND5* (complex I), *MT-CO-I*, *MT-CO-II* (complex IV), and *MT-ATP6* (complex V) [48,51,52,53]. These deletions impair the mitochondria’s ability to produce energy efficiently, particularly in high-energy-demanding tissues like the brain, heart, and muscles. In addition, other cellular mechanisms, including ROS overproduction, protein synthesis inhibition, myelin vacuolation, demyelination, autophagy, apoptosis, and the involvement of lipid rafts and oligodendrocytes in KSS pathogenesis, have also been proposed.

### 2.8. Mitochondrial Encephalomyopathy, Lactic Acidosis, and Stroke-like Episodes—MELAS

Mitochondrial encephalomyopathy, lactic acidosis, and stroke-like episodes (MELAS) are a combination of a rare mitochondrial disorder primarily caused by pathogenic variants in mtDNA. The frequency of MELAS in the general population varies significantly between 0.6 and 16 cases per 100,000 individuals, depending on the region [54]. The disorder typically presents in childhood or early adulthood, although later onset has been reported. The hallmark features include stroke-like episodes that often occur before the age of 40, causing temporary neurological deficits such as seizures, muscle weakness, and multiple ophthalmic defects. Ophthalmic defects are common in MELAS and can significantly impact vision. Key ophthalmic manifestations include: pigmentary retinopathy, macular degeneration, retinal atrophy, optic atrophy, cataracts, ptosis, ophthalmoplegia, visual field defects, nystagmus, photophobia, and progressive external ophthalmoplegia (PEO).

Other common symptoms are lactic acidosis, recurrent headaches, hearing loss, exercise intolerance, and diabetes mellitus [55,56]. The identification of mtDNA pathogenic variants in MELAS is critical for diagnosis and genetic counseling, as it follows a maternal inheritance pattern. The m.3243A>G pathogenic variant in the *MT-TL1* gene, which encodes tRNA for leucine, is the most frequently associated with MELAS, found in up to 80% of cases [57]. Other mtDNA pathogenic variants linked to the disorder include those in the *MT-ND5*, *MT-TH*, and *MT-TK* genes. Pathogenic variants, such as those in the *MT-ND5* gene, including m.13513G>A and m.13094T>C, contribute to 10% of cases [58]. These pathogenic variants contribute to the characteristic increased lactic acid accumulation, stroke-like episodes, muscle weakness, and ocular defects, which are central to the disease’s pathogenesis. In addition, the presence of angiopathy, either alone or in combination with reduced nitric oxide, has also been proposed to contribute to multi-organ involvement in MELAS syndrome [56].

### 2.9. Myoclonic Epilepsy with Ragged Red Fibers—MERRF

Myoclonic epilepsy with ragged red fibers (MERRF) is a rare maternally inherited disorder, with an estimated prevalence of around 1 in 400,000 people. The clinical features of MERRF include myoclonus, generalized seizures, ataxia, muscle weakness, hearing loss, and the presence of ragged red fibers in muscle biopsies, a hallmark of mitochondrial myopathy [40,59,60]. Ophthalmic symptoms are not the most prominent feature of MERRF, but they can occur in some patients. These may include optic atrophy, retinopathy, ptosis, ophthalmoplegia, nystagmus, and cataract. The disease manifestations may differ within the same family [61]. Symptoms typically appear in childhood or adolescence but can vary depending on the degree of heteroplasmy in cells, with higher levels of mutated mtDNA leading to more severe symptoms. MERRF is primarily caused by pathogenic variants in mtDNA, particularly affecting the tRNA genes. The most common pathogenic variant associated with MERRF is m.8344A>G (*MT-TK*), which accounts for approximately 80% of cases [40,59]. Additional pathogenic variants, such as m.8356T>C, m.8363G>A, m.8315A>C, m.3255G>A, and m.3252A>G, have also been identified, which contribute to the clinical variability of MERRF [62,63,64]. While there is no cure, early identification of mtDNA pathogenic variants is essential for diagnosis, genetic counseling, and potential future therapeutic interventions to correct mitochondrial dysfunction.

### 2.10. Non-Arteritic Anterior Ischemic Optic Neuropathy—NAION

Non-arteritic anterior ischemic optic neuropathy (NAION) is a significant cause of vision loss, particularly in older adults. It has an estimated annual incidence rate of 2.3 to 10.2 per 100,000 people, which may vary with population, being more prevalent in individuals of European descent and lowest in Blacks and Asians [65,66]. NAION typically affects individuals over 50 years old and is characterized by unilateral optic disc edema and sudden, painless vision loss [65]. Early onset and bilateral involvement are noted in familial NAION cases [67]. The exact etiology of NAION is multifactorial, but several systemic and local factors can contribute to its development. Studies have explored the potential genetic underpinnings of NAION with a primary focus on mtDNA pathogenic variants. The common mitochondrial pathogenic variants associated with NAION include the m.4132G>A variant and the m.9957T>C pathogenic variant [68,69]. The m.4132G>A pathogenic variant in the NADH dehydrogenase subunit 1 gene (*ND1*) was found in a familial NAION pedigree, where affected individuals exhibited classic NAION features [68]. This pathogenic variant was not detected in non-familial NAION patients, indicating its potential specificity to familial cases [67]. The m.9957T>C pathogenic variant has been associated with NAION in a patient who also experienced seizures, although it was not linked to MELAS [69]. Functional testing revealed significant mitochondrial dysfunction, suggesting that this pathogenic variant may contribute to optic nerve disease [69]. While these and other mtDNA pathogenic variants highlight a genetic component in NAION, the overall role of mitochondrial pathogenic variants in the broader NAION population still remains unclear, as many patients lack these specific pathogenic variants [70,71,72].

### 2.11. Primary Open-Angle Glaucoma—POAG

Primary open-angle glaucoma (POAG) is a multifactorial optic neuropathy with characteristic clinical findings of elevated intraocular pressure, progressive retinal ganglion cell (RGC) death, and optic nerve degeneration that corresponds to patterns of visual field loss. Although several genes and single nucleotide polymorphisms are associated with POAG, the underlying cause in most cases remains unexplained [73]. Several reports strongly support the role of oxidative stress and mitochondrial dysfunction in glaucoma pathogenesis [74,75].

Abu-Amero et al. were the first to demonstrate the association of potentially pathological mtDNA sequence changes and clustering of mitochondrial variants in Complex I in a Saudi Arabian POAG cohort. Their study also showed a reduction in mitochondrial respiration and increased mtDNA copy number in this cohort [74]. Several subsequent studies have corroborated the role of mitochondrial abnormalities in glaucoma in different populations [76,77,78,79,80]. Likewise, a similar clustering of mitochondrial variants in Complex I has also been observed in other optic neuropathies like LHON [15] and mitochondrial disorders like MERRF and MELAS [81]. Furthermore, Collins and colleagues have identified several mtDNA pathogenic variants with POAG in African Americans, including m.6150G>A, m.6253C>T, and m.6480G>A in *MT-CO1*, and m.2220A>G in *MT-RNR2* associated. These variants, particularly in haplogroups L1c2 and L2, were linked to increased POAG risk in this ethnicity [82] and have also been reported as risk factors in Saudi POAG patients [83]. A recent study from North India identified pathogenic mtDNA variants in POAG patients, including m.3880G>A in *ND1*, m.4852T>A in *ND2*, and m.8250G>A in *COX2*, associated with reduced cytochrome c oxidase activity and increased oxidative stress, contributing to disease severity and accounting for 32% of POAG patients harboring these changes [84]. Investigating mitochondrial genetics is critical for advancing POAG research. It may open avenues for new therapeutic approaches focused on protecting mitochondrial function and preventing RGC apoptosis in populations with a higher genetic predisposition to the disease.

### 2.12. Age-Related Macular Degeneration—AMD

Age-related macular degeneration (AMD) is a complex eye disease causing blindness in millions of elderly globally [85]. The main clinical symptom of AMD is the impairment of central vision. Oxidative stress-related damage to RPE is a critical early event in AMD initiation that eventually leads to loss of central vision due to damage to the macular, which is the hallmark of AMD [86]. Several mtDNA pathogenic variants, particularly in genes involved in OXPHOS, have been associated with AMD, suggesting a strong link between mitochondrial dysfunction and disease progression of AMD [87,88].

Common mtDNA pathogenic variants identified in AMD include changes in the *MT-ND2* (m.4917A>G), *MT-ND4* (m.11812A>G, m.11778G>A), *MT-ND6* (m.14233A>G), and *MT-ATP6* genes (m.8993T>G), which are involved in the function of the electron transport chain [89,90]. For example, the *MT-ND2* (m.4917A>G) pathogenic variant, located in a complex I subunit gene, is an independent predictor of AMD [89]. Pathogenic variants in the *MT-ND4* (m.11812A>G) and *MT-ND6* genes (m.14233A>G), both located in respiratory complex I, exhibit a 2.5-fold greater risk of advanced AMD [90]. Similarly, the *MT-ND4* (m.11778G>A) pathogenic variant, which is also known for its role in LHON, has been found in some AMD patients, particularly those with severe forms of the disease [11]. Pathogenic variants in *MT-ATP6* (m.8993T>G), another gene critical for ATP production, have also been reported in AMD, further highlighting the role of mitochondrial energy deficits in retinal degeneration [31]. Recently, Atilano et al., using NGS, have identified specific heteroplasmic variants that are more prevalent in AMD patients, suggesting potential biomarkers for the disease [91]. Notably, two variants, such as m.13095T>C and m.13105A>G, have been associated with AMD, indicating their relevance in disease progression [91].

Furthermore, specific haplogroups with mtDNA have been associated with AMD, including J, H, T, and U [90,92,93,94]. While J and U are associated with soft drusen and retinal pigment abnormalities [92], H haplogroups have been protective against AMD [94]. In addition, non-coding mtDNA D-loop pathogenic variants have shown a strong association with late-stage AMD patients [93].

Understanding the role of mtDNA pathogenic variants in AMD is crucial, as it provides insights into the underlying mechanisms driving RPE degeneration and AMD progression. Identifying individuals with specific mitochondrial pathogenic variants may enable the early detection of high-risk patients [95]. Furthermore, targeting mitochondrial pathways could lead to novel therapeutic strategies to preserve mitochondrial function and slow AMD progression [96].

**Table 1:** Mitochondrial DNA (mtDNA) genetic variants associated with ophthalmic diseases.

Locus	Allele	Amino Acid Change	Plasmy Status	Disease	Pathogenicity Classification	Study Reference No.
*ND2*	m.4917A>G	N150D	Homoplasmy	ARMD, NAION, LHON	[P]	[89,97]
*ND4*	m.11812A>G	L351L		ARMD		[98]
*ND6*	m.14233A>G	D147D				
*ND1*	m.3688G>A	A128T	Heteroplasmy	Leigh Disease	[LP]	[21]
	m.3890G>A	R195Q				
*ATP6*	m.8851T>C	W109R	Homoplasmy		[VUS]	
	m.9176T>C	L217P			[P]	
	m.9176T>G	L217R			[LP]	
	m.9191T>C	L222P	Heteroplasmy			
*COIII*	m.9537_9538insC	Frameshift				
*ND3*	m.10158T>C	S34P	Homoplasmy	Leigh Disease, MELAS	[P]	
	m.10191T>C	S45P	Heteroplasmy	Leigh Disease		
	m.10197G>A	A47T	Homoplasmy			
	m.10254G>A	D66N	Heteroplasmy		[LP]	
*ND4*	m.11777C>A	R340S				
*ND5*	m.12706T>C	F124L				
	m.13514A>G	D393G		Leigh Disease, MELAS		
*ND6*	m.14459G>A	A72V	Homoplasmy	Leigh Disease	[P]	
	m.14487T>C	M63V	Heteroplasmy			
*ND1*	m.3635G>A	S110N		LHON	[P]	
	m.3700G>A	A132T			[VUS]	
	m.3733G>A	E143K	Homoplasmy			
*ND5*	m.13051G>A	G239S	Heteroplasmy			
*ND6*	m.14482C>A	M64I	Homoplasmy		[LP]	
	m.14482C>G	M64I				
	m.14484T>C	M64V			[P]	
	m.14495A>G	L60S	Heteroplasmy		[LP]	
	m.14568C>T	G36S				
	m.14597A>G	I26T				
*ND1*	m.3460G>A	A52T	Homoplasmy	LHON, LHON-like disease	[P]	
	m.4171C>A	L289M		LHON, LHON-like disease, Leigh-like phenotype	[VUS]	
*COI*	m.6261G>A	A120T		LHON-like disease	[P]	[99]
*COII*	m.7623C>T	T13I				
*ATP6*	m.8836A>G	M104V				
*COIII*	m.9660A>C	M152L				
*ND4L*	m.10543A>G	H25R	Heteroplasmy			
	m.10591T>G	F41C				
	m.10663T>C	V65A		LHON, LHON-like disease	[LP]	[21]
*ND4*	m.11778G>A	R340H	Homoplasmy		[P]	
	m.11874C>A	T372N		LHON-like disease	[P]	[99]
*ND5*	m.12782T>G	I149S	Heteroplasmy			
	m.13379A>G	H348R		LHON, LHON-like disease	[VUS]	[21]
*ND1*	m.3376G>A	E24K	Homoplasmy	LHON /MELAS overlap		
*ND5*	m.13094T>C	V253A		LHON, MELAS	[P]	
	m.13513G>A	D393N	Heteroplasmy	LHON/MELAS Overlap Syndrome, MELAS, Leigh Disease		
*ND1*	m.3481G>A	E59K		MELAS	[LP]	
	m.3946G>A	E214K	Homoplasmy			
*CYB*	m.14787_14790del	Frameshift	Heteroplasmy			
*ND6*	m.14453G>A	A74V	Heteroplasmy	MELAS, Leigh Disease		
*ND1*	m.3697G>A	G131S	Homoplasmy			
tRNA Lysine	m.8344A>G	N/A	Heteroplasmy	MERRF	[P]	[21,59]
	m.8356T>C	N/A				
	m.3243A>G	N/A		MERRF, MELAS, Leigh Disease		[21,59,100]
	m.3255G>A	N/A		MERRF		[21,59]
	m.3291T>C	N/A		MERRF, MELAS		
	m.8363G>A	N/A		MERRF, Leigh Disease		
*ND2*	m.4762T>C	I98T	Homoplasmy	NAION		[97]
	m.5308C>A	Thr>Asn	Heteroplasmy			
*COI*	m.6335C>A	Asp>Glu	Homoplasmy			
	m.7308A>G	I469V				
*COIII*	m.9957T>C	F251L				
	m.9961T>G	Leu>Arg	Heteroplasmy			
*ND4*	m.11337A>G	N193S	Homoplasmy			
*ND5*	m.12340A>C	Thr>Pro				
	m.13834A>G	T500A				
*ND6*	m.14480A>G	V>A				
*CYTB*	m.14870A>G	I42V				
*ND1*	m.4216T>C	Y304H		NAION, LHON		
*ND5*	m.13708G>A	A458T				
*CYTB*	m.15257G>A	D171N				
	m.15674T>C	S310P		NAION, LHON-like disease, POAG		[74,97,99]
*ATP6*	m.8618_8619insT	Frameshift	Heteroplasmy	NARP	[LP]	[21]
	m.8993T>C	L156P		NARP, Leigh Disease, MILS	[P]	
	m.8993T>G	L156R	Homoplasmy			[21,101]
	m.9185T>C	L220P		NARP-like disease, Leigh Disease		[21]
*COII_ND5*	m.7983_13983del	Frameshift	Heteroplasmy	Pearson-Ptosis	[P]	[102]
*ND1*	m.3574C>A	P90T	Homoplasmy	POAG		[74]
	m.3715G>C	A137P				
	m.3903C>A	D199E				
*ND2*	m.4936C>A	T156N	Heteroplasmy			
*COI*	m.6186C>A	P95T	Homoplasmy			
	m.6219C>A	P106T				
	m.6391A>T	N163I				
	m.6459T>C	W186R				
	m.6877C>A	A325D				
*COII*	m.7827T>C	L81P				
*ATP 8*	m.8516T>A	W51X	Heteroplasmy			
*ATP 6*	m.9030C>A	H168Q	Homoplasmy			
*COIII*	m.9244C>A	P13H				
	m.9277C>A	A24D				
tRNA glycine	m.10053A>C	N/A	Heteroplasmy			
*ND3*	m.10135A>G	Q26R				
*ND4*	m.10791T>A	L11X				
	m.10920C>G	P54R	Homoplasmy			
	m.11768A>C	T337P				
	m.11867C>A	P370T				
*ND5*	m.12359C>A	T8N				

N/A: Indicates “Not Applicable” because these genes do not encode proteins. [P]: Indicates “Pathogenic’’ supported by strong and reproducible evidence from multiple independent studies. [LP]: Indicates “Likely Pathogenic’’ with significant but not definitive evidence supporting its role in disease. [VUS]: Indicates “Variant of Uncertain Significance’’ has been confirmed as existing in the population, but its clinical significance is unclear. [Homoplasmy] refers to a uniform population of mitochondrial DNA (mtDNA) within a cell, where all mtDNA molecules are identical. [Heteroplasmy] occurs when a cell contains a mixture of normal and mutated mtDNA, leading to variable expression of mitochondrial disorders.

## 3. Clinical Relevance and Limitations

The review holds significant clinical relevance and offers valuable insights for clinicians, geneticists, and researchers. Unlike the existing literature on mtDNA mutations in ophthalmic diseases that may focus on a single disease or mutation type, this review uniquely brings together both common and rare mtDNA variants across a wide range of eye-related disorders, making it a valuable reference for clinicians and researchers alike. The compilation of well-established mtDNA variants linked to conditions such as LHON, CPEO, and LS will facilitate genetic diagnosis and risk assessment and improve genetic counseling. Additionally, it explores emerging evidence of mtDNA’s association in multifactorial diseases like AMD, POAG, and NAION, highlighting new areas for research. The enhanced understanding of the genetic basis of eye diseases directly linked to pathogenic variants of mtDNA will support the development of precision medicine, guiding future advancements in mitochondrial-targeted therapies in ophthalmology. However, as the review primarily focuses on mtDNA pathogenic variants, certain limitations would need further exploration. Nuclear-encoded mitochondrial genes and DNA mutations also play a crucial role in mitochondrial dysfunction and related eye diseases, so expanding future studies to include this genetic information would provide a more comprehensive view. Furthermore, with technological advances in genomics, new mtDNA variants are rapidly discovered, and the future literature will need to integrate these findings to document the latest developments. Moreover, therapeutic options were not extensively discussed, as the primary focus was on genetic aspects of the discussed eye conditions. Future reports could address the emerging mitochondrial-targeted therapeutic avenues and clinical trials that may hold significant potential in treating and managing these disorders.

## 4. Conclusions

MtDNA pathogenic variants are integral to the pathogenesis of a diverse array of ocular diseases, reflecting the pivotal role of mitochondrial function in maintaining visual health. This review underscores the diversity and complexity of mtDNA pathogenic variants implicated in disorders such as LHON, CPEO, KSS, MELAS, and MERRF, each associated with distinct clinical manifestations. One of the most important findings highlighted in this review is the association of primary LHON mutations (m.3460G>A, m.11778G>A, m.14484T>C) with mitochondrial dysfunction, leading to RGC degeneration and optic neuropathy. Another notable discovery is the involvement of large-scale mtDNA deletions in CPEO, contributing to progressive muscle weakness and ophthalmoplegia. These findings underscore the critical role of mtDNA mutations in maintaining ocular health and the need for future studies to explore mitochondrial-targeted therapies. Emerging research suggests that mtDNA pathogenic variants also play a role in complex, multifactorial conditions, such as NAION, POAG, and AMD. For example, mtDNA mutations in *MT-ND2* and *MT-ND4* genes are linked to oxidative stress and retinal pigment epithelium damage, and may contribute to AMD. However, further research is necessary to deepen our understanding of genotype-phenotype correlations, particularly in complex multifactorial diseases. Moreover, further studies to investigate the interactions between mtDNA variants, nuclear genes, and environmental factors will be critical to making these findings more clinically significant. In conclusion, the review offers valuable insights into the genetic basis of mitochondrial-related eye diseases and highlights the need for continued research into the mechanisms, diagnostic strategies, and therapeutic options for mitochondrial-related eye diseases.

## Abbreviations

The following abbreviations are used in this manuscript:

ARMD	Age-Related Macular Degeneration
CPEO	Chronic Progressive External Ophthalmoplegia
CPEO+	Chronic Progressive External Ophthalmoplegia with additional features
KSS	Kearns-Sayre Syndrome
LHON	Leber’s Hereditary Optic Neuropathy
LHON+	Leber’s Hereditary Optic Neuropathy with additional features
MELAS	Mitochondrial Encephalomyopathy, Lactic Acidosis, and Stroke-like Episodes
MERRF	Myoclonic Epilepsy with Ragged-Red Fibers
MILS	Maternally Inherited Leigh Syndrome
NAION	Non-Arteritic Anterior Ischemic Optic Neuropathy
NARP	Neuropathy, Ataxia, and Retinitis Pigmentosa
PEO	Progressive External Ophthalmoplegia
PEO+	Progressive External Ophthalmoplegia with additional features
POAG	Primary Open-Angle Glaucoma
